# Metabolic signatures and risk of sarcopenia in suburb-dwelling older individuals by LC-MS–based untargeted metabonomics

**DOI:** 10.3389/fendo.2024.1308841

**Published:** 2024-06-19

**Authors:** Peipei Han, Xiaoyu Chen, Zhenwen Liang, Yuewen Liu, Xing Yu, Peiyu Song, Yinjiao Zhao, Hui Zhang, Shuyan Zhu, Xinyi Shi, Qi Guo

**Affiliations:** ^1^ Department of Rehabilitation Medicine, Shanghai University of Medicine and Health Sciences Affiliated Zhoupu Hospital, Shanghai, China; ^2^ College of Rehabilitation Sciences, Shanghai University of Medicine and Health Sciences, Shanghai, China; ^3^ Jiangwan Hospital of Shanghai Hongkou District, Shanghai University of Medicine and Health Science Affiliated First Rehabilitation Hospital, Shanghai, China

**Keywords:** LC-MS, risk, sarcopenia, untargeted metabonomics, Chinese

## Abstract

**Background:**

Untargeted metabonomics has provided new insight into the pathogenesis of sarcopenia. In this study, we explored plasma metabolic signatures linked to a heightened risk of sarcopenia in a cohort study by LC-MS-based untargeted metabonomics.

**Methods:**

In this nested case–control study from the Adult Physical Fitness and Health Cohort Study (APFHCS), we collected blood plasma samples from 30 new-onset sarcopenia subjects (mean age 73.2 ± 5.6 years) and 30 healthy controls (mean age 74.2 ± 4.6 years) matched by age, sex, BMI, lifestyle, and comorbidities. An untargeted metabolomics methodology was employed to discern the metabolomic profile alterations present in individuals exhibiting newly diagnosed sarcopenia.

**Results:**

In comparing individuals with new-onset sarcopenia to normal controls, a comprehensive analysis using liquid chromatography-mass spectrometry (LC-MS) identified a total of 62 metabolites, predominantly comprising lipids, lipid-like molecules, organic acids, and derivatives. Receiver operating characteristic (ROC) curve analysis indicated that the three metabolites hypoxanthine (AUC=0.819, 95% CI=0.711–0.927), L-2-amino-3-oxobutanoic acid (AUC=0.733, 95% CI=0.598–0.868) and PC(14:0/20:2(11Z,14Z)) (AUC= 0.717, 95% CI=0.587–0.846) had the highest areas under the curve. Then, these significant metabolites were observed to be notably enriched in four distinct metabolic pathways, namely, “purine metabolism”; “parathyroid hormone synthesis, secretion and action”; “choline metabolism in cancer”; and “tuberculosis”.

**Conclusion:**

The current investigation elucidates the metabolic perturbations observed in individuals diagnosed with sarcopenia. The identified metabolites hold promise as potential biomarkers, offering avenues for exploring the underlying pathological mechanisms associated with sarcopenia.

## Introduction

Sarcopenia, defined by the gradual decline in skeletal muscle mass, muscle strength, and physical performance (1), is closely linked to the aging process and adverse health outcomes, including disability, diabetes, metabolic syndrome, poor quality of life, and increased mortality (2). Despite the severity of its symptoms and associated side effects, the pathophysiological mechanisms driving sarcopenia remain inadequately elucidated, and current pharmacotherapies demonstrate limited efficacy (3). Therefore, there exists a pressing imperative to uncover novel, clinically significant biomarkers, delineate high-risk populations, and attain a more profound understanding of the underlying pathological mechanisms to mitigate the onset and progression of sarcopenia.

Metabonomics emerges as a potent methodology capable of furnishing intricate insights into biological pathways, pivotal genes, and mutations, thus elucidating the mechanisms underlying disease progression and revealing diagnostic biomarkers Metabonomics analysis can be performed using untargeted or targeted approaches. Most of the current metabonomics studies were targeted metabonomics analyses (4–6).

Nevertheless, while targeted metabonomics offers precision, it inherently limits the potential for discovering novel biomarkers and unveiling previously unrecognized pathways in sarcopenia development. Untargeted metabolomic analysis emerges as essential for functional research, facilitating the integration of comprehensive metabolic profiles with biological insights (7).

Several integrated studies investigating the relationship between blood and fecal untargeted metabolomics and sarcopenia have been published. These studies involve untargeted profiling to discern trait-specific or shared metabolites linked with muscle mass and strength (8), comparing the plasma metabolome (9, 10), as well as analyzing the differences in fecal metabolites (11–13). Blood metabolomics primarily reflects systemic metabolic changes and can capture metabolites related to muscle metabolism, hormonal regulation, and systemic inflammation, which are directly relevant to the pathophysiology of sarcopenia. In contrast, fecal metabolomics offers insights into gut microbiota composition and activity, which, while important for overall health, may be less directly connected to muscle metabolism. The systemic nature of blood metabolomics makes it particularly valuable for identifying biomarkers that are directly associated with muscle mass and strength, and for understanding the systemic metabolic disturbances underlying sarcopenia.

Multiple studies have proposed that the characterization of metabolic signatures holds promise as a biomarker for investigating the physical debilitation associated with sarcopenia (14). However, research on metabolomic analysis in sarcopenia remains in its incipient stages. More research is needed to identify more valuable potential markers, especially by untargeted metabonomics. Furthermore, studies using untargeted metabonomics approaches have predominantly been conducted in Western populations (8, 9, 15, 16). Asians and Westerners differ in lifestyle factors such as exercise habits, sleep quality, stress levels, etc. These lifestyle factors potentially influence metabolic processes, thereby contributing to differences in metabolomic profiles.

Data from Asian populations are scarce and predominantly derived from cross-sectional studies, which are vulnerable to reverse causation and hinder the establishment of temporal and causal relationships. To our knowledge, only two studies have been conducted on Asian populations (17, 18).

Here, the objective of this study was to pinpoint potential plasma metabolite biomarkers linked with sarcopenia. These findings will enrich our understanding of sarcopenia’s progression and may aid in identifying new molecular targets for the treatment of this condition.

## Materials and methods

### Study, setting, and design

The study design entailed a nested case-control approach derived from the Adult Physical Fitness and Health Cohort Study (APFHCS) [ChiCTR1900024880]. The APFHCS is a substantial prospective, open, and dynamic cohort study primarily examining the correlation between physical fitness and health status within a broad adult population residing in Tianjin and Shanghai, China. All participants enrolled in the National Free Physical Examination Program were recruited for comprehensive annual health assessments. Subsequently, they were instructed to fill out detailed questionnaires regarding their lifestyle and medical history. Subsequently, they were subjected to lifelong follow-up through periodic visits. The characteristics of the study participants have been delineated in our prior investigation (19).

Subjects in this study participated in September 2019 (baseline) and September 2020 (follow-up) in Shanghai. A total of 380 subjects had a plasma sample available at baseline. Participants who met any of the following conditions were excluded from the study: (1) inability to provide informed consent; (2) inability to undergo anthropometric measurements; and (3) diagnosis of sarcopenia using the AWGS criteria. Among the 380 subjects, 48 individuals diagnosed with sarcopenia in 2019 were excluded from the analysis. Subsequently, 30 subjects who developed new-onset sarcopenia in 2020 were matched with non-sarcopenic individuals based on age, sex, BMI, lifestyle, and comorbidities using propensity score matching. Nearest neighbor matching without replacement was employed in a 1:1 manner, with a caliper set at 0.02 standard deviations of the logit of the propensity score. This study was approved by the Ethics Committee of Shanghai University of Medicine and Health Sciences. All study participants voluntarily participated by providing written informed consent and completing comprehensive questionnaires encompassing demographic variables, including age, sex, lifestyle behaviors, and medical history. The measurement methods were detailed in our previous cross-sectional study (19).

### Assessment of sarcopenia

Sarcopenia was defined according to the Asian Working Group for Sarcopenia (AWGS) criteria (20), in which a person who has low muscle mass, low muscle strength and/or low physical performance was identified as having sarcopenia. Low muscle mass was classified as a relative skeletal muscle mass index (ASM/ht^2^) less than 7.0 kg/m2 and 5.7 kg/m2 in men and women, respectively; low muscle strength was defined as grip strength <28 kg or <18 kg for males and females, respectively; and low physical performance was defined as walking speed <1.0 m/s for both males and females.

Muscle mass was quantified employing direct segmental multi-frequency bioelectrical impedance analysis (BIA) (In-Body720; Biospace Co., Ltd,Seoul, Korea). Assessment of muscle strength involved measuring grip strength using a dynamometer (GRIP-D; Takei Ltd, Niigata, Japan) while standing, with the arm fully extended straight down by the side. The participant received instructions to exert maximal effort by squeezing the handle of the dynamometer for a duration of 3–5 seconds, accompanied by standard encouragement to ensure maximal performance. Following this, the measurement was repeated after a 30-second interval to allow for recovery. The usual walking speed, measured in meters per second (m/s), was utilized as an objective indicator of physical performance, conducted along a 4-meter course. The methodology for this measurement has been previously elucidated in our prior study (21).

### Sample collection and processing

Each plasma sample was procured from the study participants under fasting conditions in the morning. Subsequently, the samples were separated and preserved in freezers at a temperature of -80°C until the commencement of the metabonomics assay. Thawing of the samples was conducted at room temperature prior to analysis. Initially, 150 μl of plasma was aliquoted into a new Eppendorf tube, followed by the addition of 10 μl of L-2-chlorophenylalanine (0.3 mg/ml) dissolved in methanol as the internal standard. Subsequently, a 450-μl mixture of methanol/acetonitrile (2/1) was added and vortexed for 1 minute. The entire samples underwent extraction by ultrasonication for 10 minutes and were then stored at -20°C for 30 minutes. The resulting extract was centrifuged for 10 minutes at 4°C (13,000 rpm). A volume of 200 μl of supernatant was then subjected to drying in a freeze concentration centrifugal dryer, followed by resolubilization with 300 μl of methanol/water (1/4) and vortexing for 30 seconds. Subsequent extraction by ultrasonication for 3 minutes was performed. After thorough mixing, the samples were centrifuged at 4°C (13,000 rpm) for 10 minutes, and 150 μl of supernatant was filtered through 0.22-μm microfilters before being transferred to LC vials. The vials were stored at -80°C prior to analysis by LC-MS.

### Metabolic profiling

Plasma metabolomic profiling analysis was conducted in accordance with previously established methodologies (22). In brief, LC-MS analysis was executed utilizing an ACQUITY ultra-performance liquid chromatography (UPLC) I-Class coupled with a VION IMS QT high-resolution mass spectrometer (Waters Corporation, Milford, USA). Metabolic profiles were obtained through electrospray ionization (ESI) in both positive and negative ion modes. Sample separation was conducted using an ACQUITY UPLC BEH C18 column (Waters Corporation; particle size: 1.7 μm, dimensions: 100 × 2.1 mm) at a flow rate of 0.4 ml/min. The column temperature was held constant at 45°C, while the sample chamber temperature was maintained at 4°C, and a 1 μl injection volume was employed. The mobile phases consisted of two solutions: solution A comprised water with 0.1% formic acid, while solution B consisted of a mixture of acetonitrile and methanol in a ratio of 2:3 (vol/vol) with 0.1% formic acid. The gradient elution program was as follows: from 0 to 1 minute, the composition was 30% solution B; from 1 to 2.5 minutes, the composition increased from 30% to 60% solution B; from 2.5 to 6.5 minutes, it increased from 60% to 90% solution B; from 6.5 to 8.5 minutes, it increased from 90% to 100% solution B; at 8.5 to 10.7 minutes, the composition remained at 100% solution B; from 10.7 to 10.8 minutes, it decreased from 100% to 1% solution B; and finally, from 10.8 to 13 minutes, the composition was maintained at 1% solution B. The LC-MS system was operated under optimized conditions, including an ion source temperature of 150°C, capillary voltage set to 2.5 kV, desolvation gas flow maintained at 900 L/h, declustering potential set at 40V, collision energy at 4 eV, a mass scan range from m/z 50 to 1,000, and a scan time of 0.2 s (22).

### Data processing and analysis

The LC-MS data underwent processing using Progenesis QI version 2.3 software (Nonlinear Dynamics, Newcastle, UK) to facilitate meaningful data analysis, encompassing peak alignment, selection, normalization, and retention time (RT) correction. The resultant feature matrix comprised information on mass-to-charge ratio (m/z), RT, and peak intensities. Compound identification relied on precise m/z values, secondary fragments, and isotopic distribution, with reference to databases such as the Human Metabolome Database (HMDB) (http://www.hmdb.ca/), LIPID MAPS (version 2.3) (http://www.lipidmaps.org/), Metabolite Mass Spectral Database (METLIN) (http://metlin.scripps.edu/), and internally curated databases (EMDB) for qualitative analysis.

In order to delineate differences in metabolic profiles between the control group and individuals with newly diagnosed sarcopenia, orthogonal projection to latent structure with discriminant analysis (OPLS-DA) was utilized as a statistical approach. Simultaneously, the OPLS-DA model underwent validation using a 200-fold permutation test. This permutation test was evaluated through cross-validation, during which the correlation coefficients R2 and Q2 derived from the cross-validation procedure were examined to determine the likelihood of overfitting (23).

Variations in expression between groups were evaluated employing both multidimensional and single-dimensional analyses. The variable importance in projection (VIP) scores derived from OPLS-DA were employed to identify metabolites demonstrating significant biological disparities. Additionally, the statistical significance of these differentially expressed metabolites was confirmed through Student’s t-test. Metabolites with VIP scores exceeding 1.0 and P-values below 0.05 were considered potential biomarkers indicative of sarcopenia. The predictive accuracy of the model was assessed via the computation of the area under the receiver operating characteristic (ROC) curve (AUC). Furthermore, to elucidate the underlying mechanisms driving metabolic pathway alterations across distinct sample groups, the differentially expressed metabolites underwent metabolic pathway enrichment analysis utilizing the Kyoto Encyclopedia of Genes and Genomes (KEGG) database (http://www.kegg.jp/kegg/pathway.html).

The baseline sociodemographic characteristics of both the control and newly diagnosed sarcopenia cohorts were juxtaposed employing an independent t-test for numerical variables and the chi-squared test for categorical variables. Data exhibiting a normal distribution were depicted as the mean along with the standard deviation (SD), whereas categorical variables were delineated as proportions. Statistical assessments were conducted using SPSS version 26.0 (SPSS Incorporation, Chicago, IL, USA), with significance threshold established at p < 0.05.

## Results

### Participants

During this study, 30 new-onset sarcopenia subjects and 30 non-sarcopenia subjects (control group) were eventually incorporated into our study. The primary characteristics of the population are delineated based on the cases and controls in **Table 1**. There were no statistically significant discrepancies noted in age, gender distribution, BMI, or other pertinent indicators across the groups, implying that individuals within each group were similar in baseline characteristics.

**Table 1:** Baseline characteristics of the matched groups.

Characteristic	New-onset Sarcopenia Group(n=30)	Control Group(n=30)	*P* value
Age (years)	73.2 ± 5.6	74.2 ± 4.6	0.483
Sex			1.000
Male (%)	40.0	40.0	
Female (%)	60.0	60.0	
BMI (kg/m2)	22.7 ± 2.7	22.9 ± 2.6	0.778
MNA (score)	12.4 ± 1.6	12.9 ± 1.1	0.122
IPAQ (Met-min/wk)	5313(643–9571)	6294(1195–13440)	0.438
GDS (score)	5.6 ± 3.7	5.4 ± 3.7	0.848
Sleep quality (score)	0.37 ± 0.68	0.42 ± 0.74	0.764
Illiteracy (%)			0.299
No	76.7	90.0	
Yes	23.3	10.0	
Widowed (%)			0.209
No	70.0	86.7	
Yes	30.0	13.3	
Farming (%)			0.781
No	30.0	33.3	
Yes	70.0	66.7	
Smoking (%)			0.688
No	90.0	86.7	
Yes	10.0	13.3	
Drinking (%)			0.417
No	70.0	60.0	
Yes	30.0	40.0	
Diabetes (%)			0.145
No	79.3	93.3	
Yes	20.7	6.7	
Hypertension (%)			1.000
No	23.3	23.3	
Yes	76.7	76.7	
Hyperlipidemia (%)			0.422
No	56.7	70.0	
Yes	43.3	30.0	
Heart disease (%)			0.795
No	58.6	53.3	
Yes	41.4	46.7	

BMI, Body mass index; MNA, Mini nutritional assessment; IPAQ, International physical activity questionnaires; GDS, Geriatric depression scale.

### Multivariate statistical analysis

**Figure 1:**
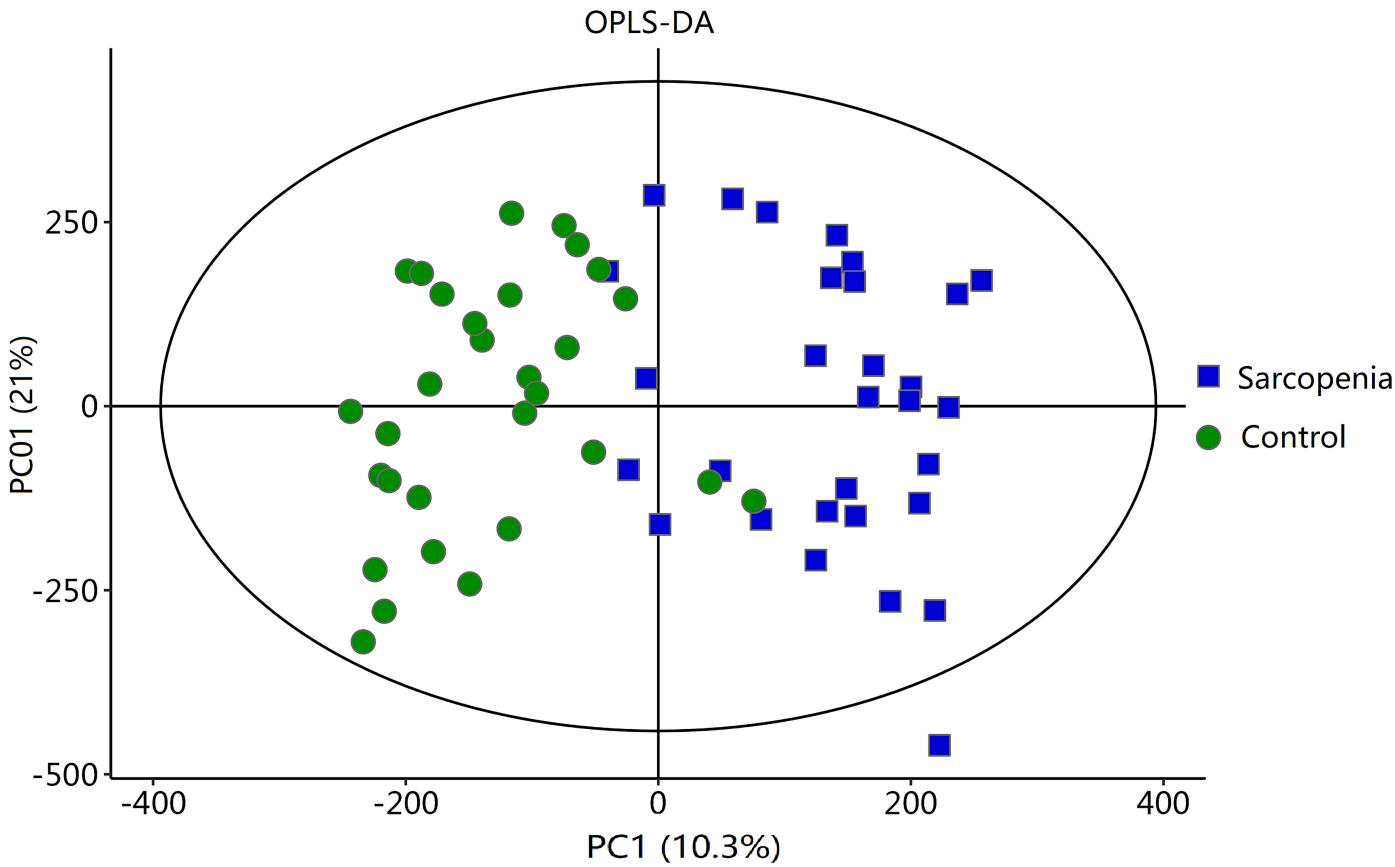
OPLS-DA score plot comparing the new-onset sarcopenia group with the matched control group.

**Figure 2:**
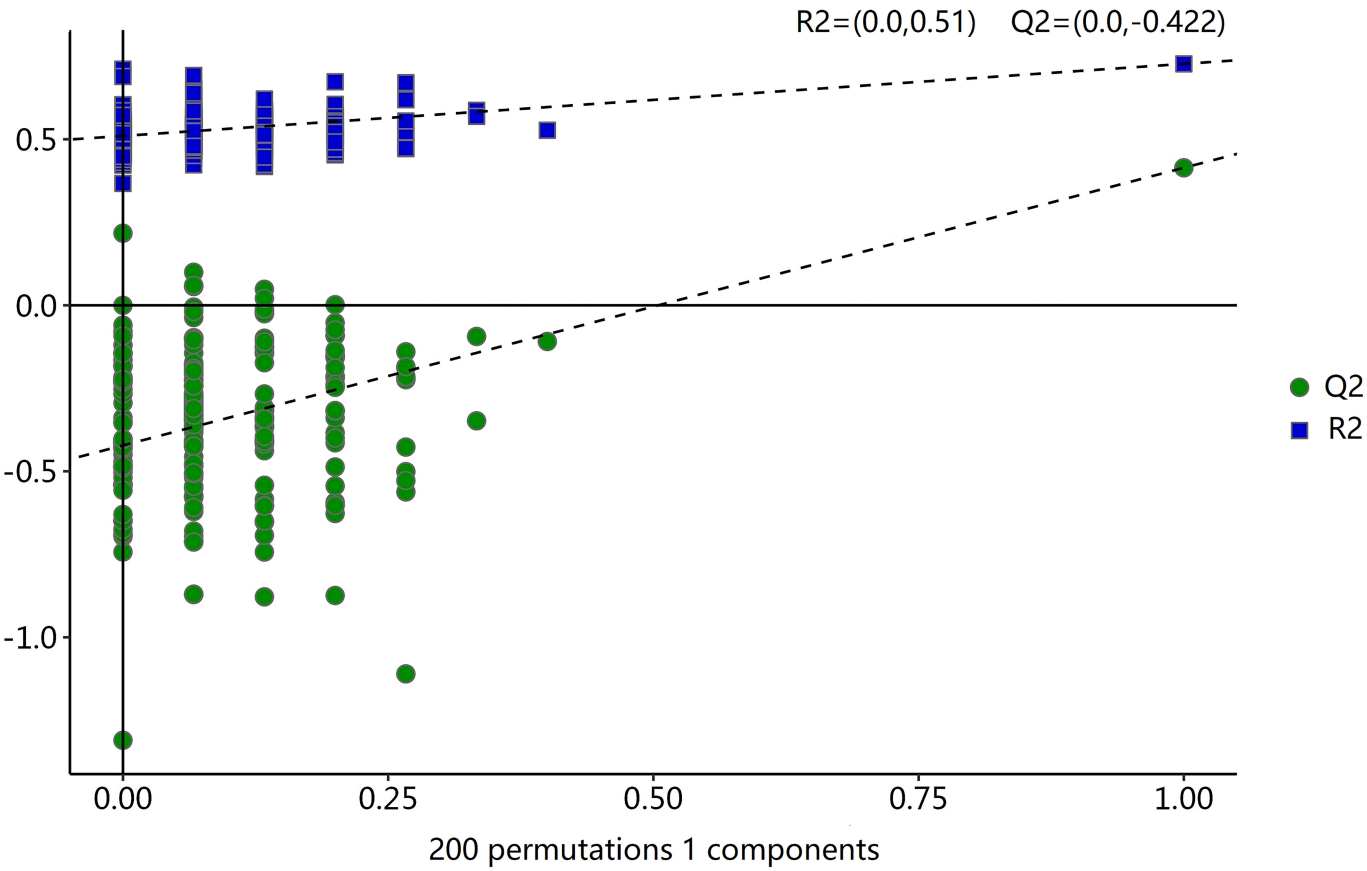
Statistical validation of the OPLS-DA model by permutation testing with 200 iterations.

To analyze the metabolic changes between the new-onset sarcopenia and matched control groups, nontargeted metabonomics analysis was performed using LC−MS. OPLS-DA serves as a suitable model for discerning distinct origins in scenarios where multiple factors may influence metabolite profiles. Consequently, we established an OPLS-DA model utilizing the metabolic spectrum, revealing an evident tendency for segregation, as illustrated in **Figure 1**. Furthermore, additional permutation tests illustrated that the model was not overfitted, with R2= (0.0, 0.51) and Q2= (0.0, -0.42) (**Figure 2**). Taken together, these findings suggest a significant metabolic alteration between the new-onset sarcopenia group and the matched control group.

### Metabolomic differences between the studied groups

**Figure 3:**
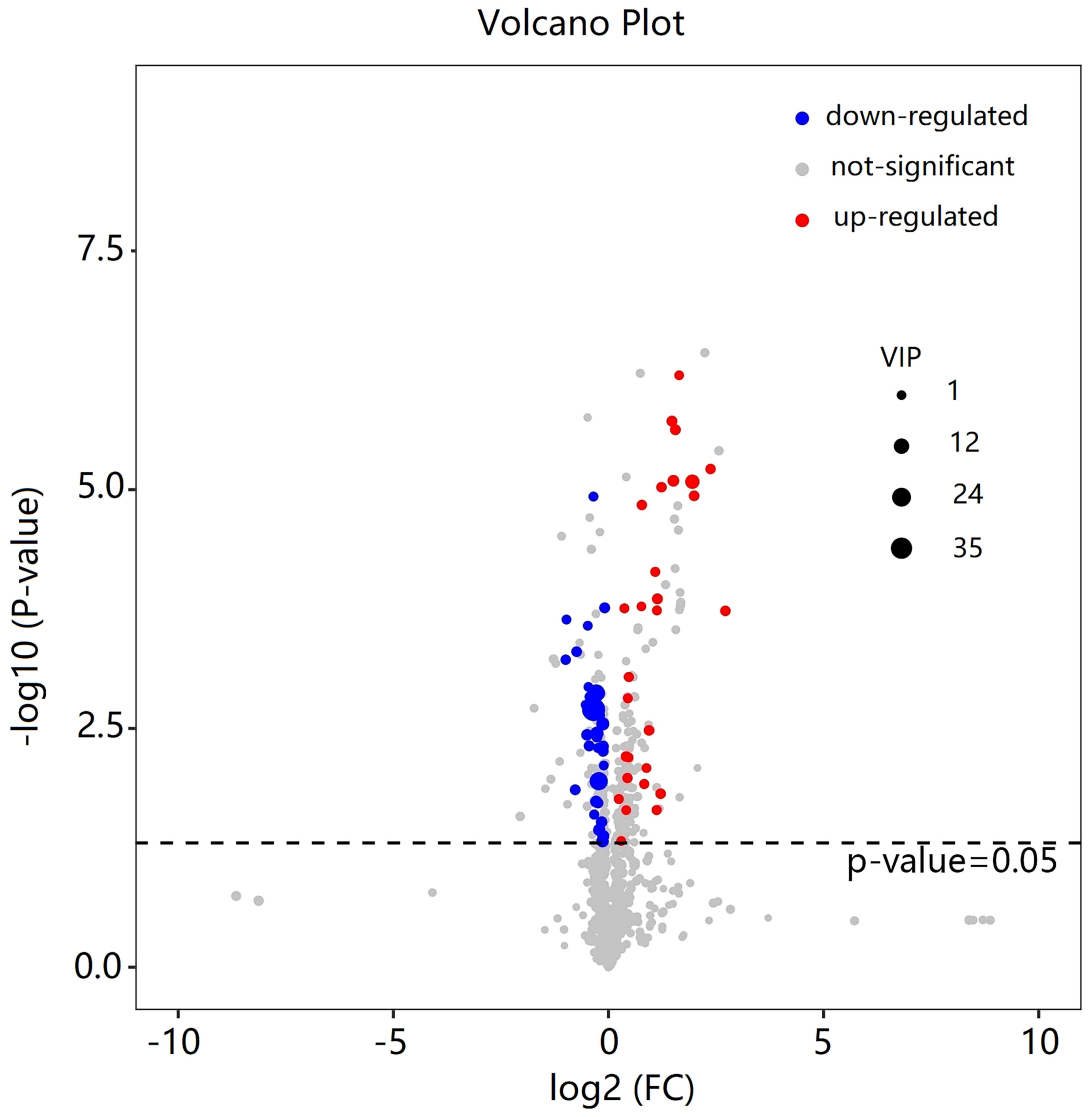
Volcano plot of the differential metabolites filtered by the univariate analysis between the new-onset sarcopenia and matched control groups.

**Figure 4:**
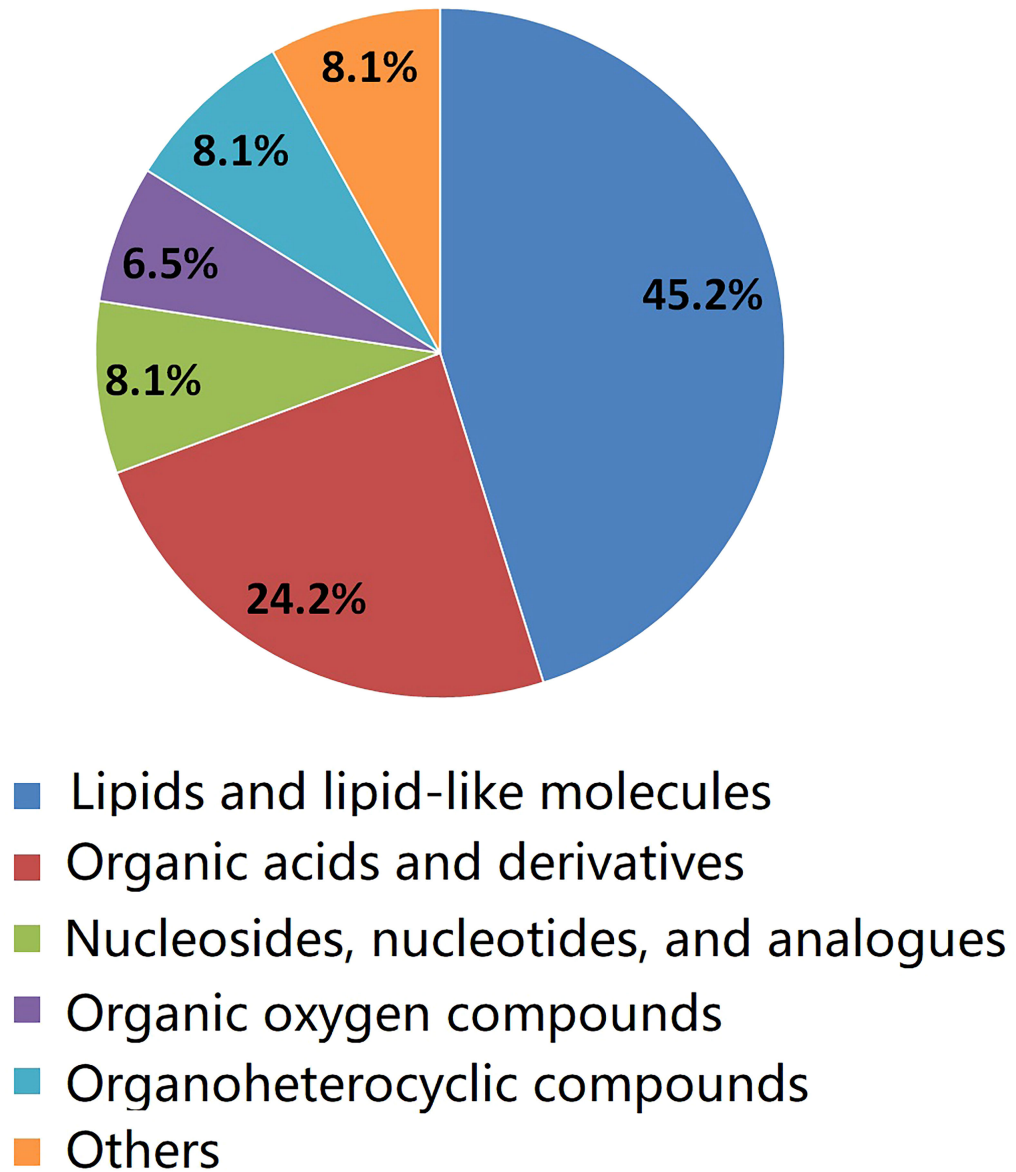
The pie chart illustrates the classification and quantity of significantly disturbed metabolites.

**Figure 5:**
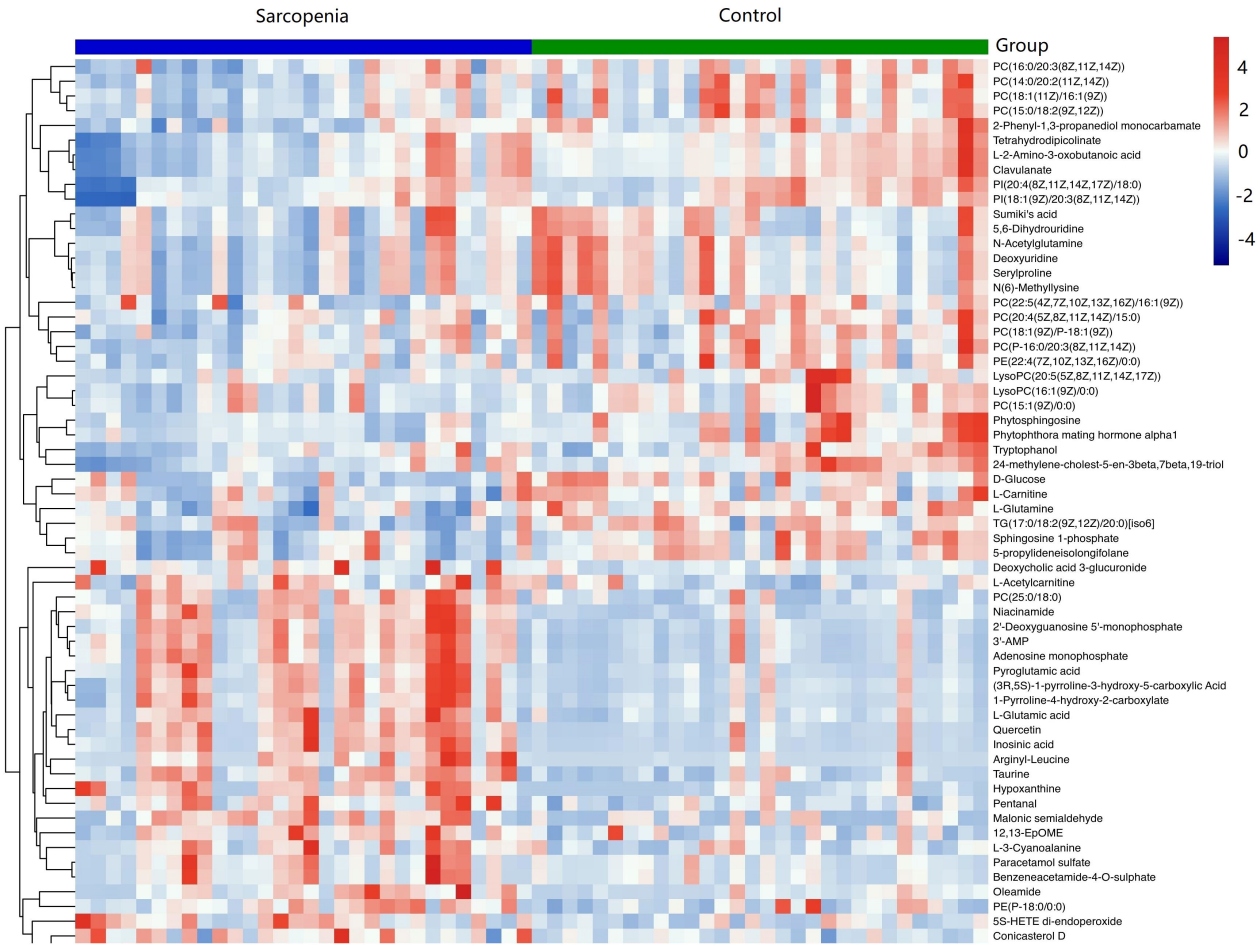
Hierarchical clustering revealed the profiles of important differential metabolites in samples from the new-onset sarcopenia and matched control groups. Blue indicates decreased levels, while red indicates increased levels.

As shown in **Table 2**, a total of 62 differentially expressed metabolites, including 34 downregulated and 28 upregulated differentially expressed metabolites, contributed significantly to the distinction between the control group and the new-onset sarcopenia group. The volcano plot displays both the p-value and fold change value (**Figure 3**), thereby substantiating the efficacy of differential metabolites. The majority of these distinct metabolites were identified as lipids and lipid-like molecules, along with organic acids and derivatives, which accounted for 45.2% and 24.2%, respectively (**Figure 4**). Hierarchical clustering was performed using the expression profiles of all metabolites demonstrating significant disparities (**Figure 5**). In this visualization, colors indicate elevated (red) or diminished (blue) levels of abundance, with intensity correlating to the respective concentration. These findings provide a more direct depiction of the associations among samples and the variations in metabolite expression across different samples.

**Table 2:** The 62 differential metabolites associated with risk of sarcopenia.

m/z	Ion mode	Metabolite	kegg	Status	VIP	p value	log2(FC)
758.569	pos	PC(14:0/20:2(11Z,14Z))	C00157	↓	44.90	0.002	-0.35
784.583	pos	PC(16:0/20:3(8Z,11Z,14Z))	C00157	↓	21.12	0.011	-0.23
802.560	neg	PC(18:1(11Z)/16:1(9Z))	C00157	↓	18.26	0.001	-0.28
137.046	pos	Hypoxanthine	C00262	↑	9.00	<0.001	1.95
235.092	pos	L-2-Amino-3-oxobutanoic acid	C03508	↓	6.45	0.004	-0.27
273.072	neg	Deoxyuridine	C00526	↓	6.01	0.003	-0.13
742.540	neg	PC(15:0/18:2(9Z,12Z))	C00157	↓	5.12	0.001	-0.32
850.560	neg	PC(22:5(4Z,7Z,10Z,13Z,16Z)/16:1(9Z))	C00157	↓	4.81	0.048	-0.14
792.586	pos	PC(18:1(9Z)/P-18:1(9Z))	C00157	↓	4.31	0.036	-0.22
160.060	pos	Sumiki’s acid	C20448	↓	3.98	0.042	-0.12
885.551	neg	PI(20:4(8Z,11Z,14Z,17Z)/18:0)	C00626	↓	3.94	0.018	-0.29
348.070	pos	2’-Deoxyguanosine 5’-monophosphate	C00362	↑	3.54	<0.001	1.51
538.315	neg	LysoPC(16:1(9Z)/0:0)	C04230	↓	3.26	0.004	-0.50
247.093	neg	Serylproline		↓	3.25	0.005	-0.12
766.541	neg	PC(20:4(5Z,8Z,11Z,14Z)/15:0)	C00157	↓	3.11	0.030	-0.16
194.082	neg	2-Phenyl-1,3-propanediol monocarbamate	C16586	↓	2.41	<0.001	-0.09
478.294	neg	PC(15:1(9Z)/0:0)		↓	2.37	0.005	-0.45
128.035	neg	(3R,5S)-1-pyrroline-3-hydroxy-5-carboxylic Acid	C04281	↑	2.35	<0.001	1.14
346.056	neg	3’-AMP	C01367	↑	2.34	<0.001	1.48
159.114	neg	N(6)-Methyllysine	C02728	↓	2.33	0.005	-0.13
378.242	neg	Sphingosine 1-phosphate	C06124	↓	2.29	0.001	-0.43
348.070	pos	Adenosine monophosphate	C00020	↑	2.24	<0.001	1.56
542.324	pos	LysoPC(20:5(5Z,8Z,11Z,14Z,17Z))	C04230	↓	2.23	0.014	-0.77
217.082	pos	Clavulanate	C06662	↓	2.19	0.004	-0.27
885.550	neg	PI(18:1(9Z)/20:3(8Z,11Z,14Z))	C00626	↓	2.10	0.019	-0.24
318.300	pos	Phytosphingosine	C12144	↓	2.09	<0.001	-0.74
812.581	neg	PC(P-16:0/20:3(8Z,11Z,14Z))		↓	2.05	0.012	-0.28
347.042	neg	Quercetin	C00389	↑	2.04	<0.001	1.99
249.054	pos	Paracetamol sulfate		↑	1.92	0.015	1.21
204.123	pos	L-Acetylcarnitine	C02571	↑	1.92	0.006	0.41
288.203	pos	Arginyl-Leucine		↑	1.89	<0.001	2.72
130.050	pos	Pyroglutamic acid	C01879	↑	1.89	<0.001	1.23
175.025	neg	Malonic semialdehyde	C00222	↑	1.86	0.003	0.94
124.007	neg	Taurine	C00245	↑	1.81	<0.001	0.78
347.040	neg	Inosinic acid	C00130	↑	1.81	<0.001	2.37
203.052	pos	D-Glucose	C00221	↓	1.79	<0.001	-0.99
162.112	pos	L-Carnitine	C00318	↓	1.76	0.036	-0.22
132.077	pos	L-3-Cyanoalanine	C02512	↑	1.62	0.010	0.44
184.073	pos	Tryptophanol	C00955	↓	1.50	0.025	-0.33
249.054	pos	Benzeneacetamide-4-O-sulphate		↑	1.48	0.023	1.12
567.318	neg	Deoxycholic acid 3-glucuronide	C03033	↑	1.45	0.012	0.83
189.087	pos	Tetrahydrodipicolinate	C03972	↓	1.42	0.002	-0.20
247.092	pos	5,6-Dihydrouridine		↓	1.41	0.043	-0.11
425.215	pos	5S-HETE di-endoperoxide		↑	1.37	0.001	0.48
130.050	pos	1-Pyrroline-4-hydroxy-2-carboxylate	C04282	↑	1.34	<0.001	1.09
295.228	neg	12,13-EpOME	C14826	↑	1.34	0.017	0.24
145.062	neg	L-Glutamine	C00064	↓	1.30	<0.001	-0.35
489.359	neg	Conicasterol D		↑	1.29	0.002	0.45
104.107	pos	Pentanal		↑	1.26	<0.001	0.37
910.722	pos	PC(25:0/18:0)		↑	1.25	<0.001	1.13
528.310	neg	PE(22:4(7Z,10Z,13Z,16Z)/0:0)		↓	1.23	0.005	-0.25
362.326	pos	Phytophthora mating hormone alpha1		↓	1.18	<0.001	-0.98
923.803	pos	TG(17:0/18:2(9Z,12Z)/20:0)[iso6]		↓	1.17	0.002	-0.53
453.334	pos	24-methylene-cholest-5-en-3beta,7beta,19-triol		↓	1.15	<0.001	-0.48
445.332	neg	25(OH)D3	C01561	↑	1.14	0.048	0.30
282.279	pos	Oleamide	C19670	↑	1.13	0.008	0.88
264.269	pos	5-propylideneisolongifolane		↓	1.10	0.001	-0.47
466.330	pos	PE(P-18:0/0:0)		↑	1.09	0.023	0.41
187.072	neg	N-Acetylglutamine		↓	1.08	0.008	-0.11
123.055	pos	Niacinamide	C00153	↑	1.06	<0.001	1.64
517.390	neg	Theonellasterol D		↑	1.03	0.006	0.47
146.046	neg	L-Glutamic acid	C00302	↑	1.01	<0.001	0.77

Relative concentrations compared to control group: ↑, upregulated; ↓, downregulated.

### Evaluation of the metabolite panel for the diagnosis of sarcopenia

Univariate ROC curve analysis was conducted for the top 10 potential biomarkers. Among these, five metabolites exhibited areas under the ROC curve (AUCs) of at least 0.7 (**Table 3**). Notably, the three most discriminative metabolites with the highest accuracy were identified as hypoxanthine (AUC=0.819, 95% CI=0.711–0.927), L-2-amino-3-oxobutanoic acid (AUC=0.733, 95% CI=0.598–0.868), and PC(14:0/20:2(11Z,14Z)) (AUC= 0.717, 95% CI=0.587–0.846). We conducted a further analysis of the correlation between these three metabolites and the components of sarcopenia (muscle mass, grip strength, and walking speed). The results indicated that hypoxanthine is negatively correlated with walking speed, whereas L-2-amino-3-oxobutanoic acid and PC (14:0/20:2(11Z,14Z)) are positively correlated with both grip strength and walking speed.

**Table 3:** The AUC values for metabolites.

Metabolite	AUC	95%CI	P
Hypoxanthine	0.819	0.711–0.927	<0.001
L-2-Amino-3-oxobutanoic acid	0.733	0.598–0.868	0.002
PC(14:0/20:2(11Z,14Z))	0.717	0.587–0.846	0.004
Deoxyuridine	0.713	0.581–0.846	0.005
PC(15:0/18:2(9Z,12Z))	0.710	0.578–0.842	0.005
PC(18:1(11Z)/16:1(9Z))	0.693	0.559–0.828	0.010
PC(16:0/20:3(8Z,11Z,14Z))	0.687	0.550–0.823	0.013
PC(22:5(4Z,7Z,10Z,13Z,16Z)/16:1(9Z))	0.667	0.526–0.807	0.071
Sumiki’s acid	0.666	0.527–0.804	0.070
PC(18:1(9Z)/P-18:1(9Z))	0.643	0.502–0.784	0.071

### Metabolic enrichment analysis and pathway analysis

**Figure 6:**
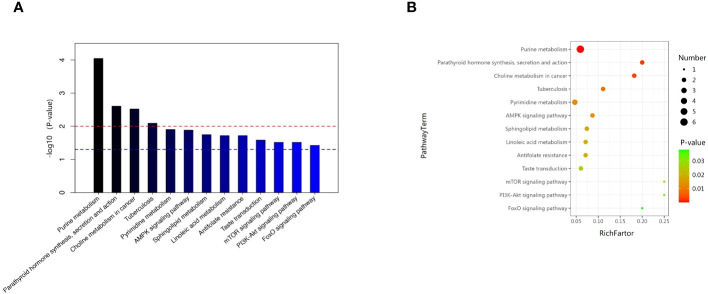
**(A)** Pathway analysis indicates that sphingolipid metabolism is the most statistically enriched pathway. **(B)** Metabolic pathway analysis based on plasma metabolites.

We examined the metabolic pathways potentially implicated in the observed changes in metabolic profiles associated with new-onset sarcopenia. Through pathway enrichment analysis, we demonstrated that “purine metabolism”, “parathyroid hormone synthesis, secretion and action”, “choline metabolism in cancer”, and “tuberculosis” emerged as the most significantly perturbed pathways in new-onset sarcopenia (**Figure 6**).

## Discussion

In this prospective study, we systematically present the results of our longitudinal investigation into the associations between plasma metabolites and the onset of sarcopenia utilizing LC-MS-based untargeted metabonomics. This investigation includes a nested case–control study that specifically targets new-onset sarcopenia within a cohort study. We identified 62 metabolites that were correlated with an increased risk of developing new-onset sarcopenia. Among these, lipids and lipid-like molecules, as well as organic acids and derivatives, emerged as the predominant altered metabolites in individuals with new-onset sarcopenia compared to the control group. Currently, the few existing studies on non-targeted metabolomics of sarcopenia have yielded inconsistent results (8, 9, 15–18). The reason for the discrepancy is likely due to differences in lifestyle, such as exercise, sleep, diet, and psychological factors, as well as variations in populations and ethnicity. The top three metabolites demonstrating the highest discriminatory power between the groups were hypoxanthine, L-2-amino-3-oxobutanoic acid, and PC(14:0/20:2(11Z,14Z)). In summary, our study delineates a panel of 62 metabolic signatures, which can be broadly classified into four pathways: (1) purine metabolism; (2) parathyroid hormone synthesis, secretion and action; (3) choline metabolism in cancer; and (4) tuberculosis.

### Relationship between the top three metabolites and sarcopenia

In this study, it was discovered that hypoxanthine levels were significantly elevated in the new-onset sarcopenia group compared to the control group, rendering it the most discriminative metabolite. Similarly, the studies by Shida T et al. (24) and Zhou J et al. (12) also observed higher hypoxanthine levels in the sarcopenia group compared to the non-sarcopenia group. Animal models of diabetes corroborate this hypothesis, as metabonomics analysis of atrophied quadriceps femoris muscles demonstrated significantly higher levels of hypoxanthine (25). In addition, previous investigations have reported that increases in circulating AdN degradation products are distinctive features of human sarcopenia (26–28), and hypoxanthine was one of the important AdN degradation products. Given that hypoxanthine originates from inosine metabolized by purine nucleoside phosphorylase activity (29), the observed elevations in the levels of inosine, xanthine, and hypoxanthine collectively indicate signs of oxidative stress within the muscle tissues of mice. Oxidative stress is recognized to play a pivotal role in the pathogenesis of sarcopenia (30).

L-2-amino-3-oxobutanoic acid emerged as the second metabolite with the highest discriminatory power between the groups. To our knowledge, there have been no previous reports of direct observations of L-2-amino-3-oxobutanoic acid in the context of sarcopenia. Previous research has indicated that L-2-amino-3-oxobutanoic acid is a downstream metabolite of glycine metabolism (31), and glycine has been shown in rodent models to reduce plasma insulin levels and decrease fat mass (32). Thus, we hypothesize that L-2-amino-3-oxobutanoic acid may be involved in the pathogenesis of sarcopenia through the modulation of glycine metabolism, potentially affecting insulin sensitivity, antioxidative, and anti-inflammatory capacities. However, we also note that only a few studies have linked L-2-amino-3-oxobutanoic acid to other metabolic diseases such as diabetes (33) and liver injury (31). Therefore, further research is warranted to elucidate the exact role and mechanisms of L-2-amino-3-oxobutanoic acid in the pathogenesis of sarcopenia.

In addition to hypoxanthine and L-2-amino-3-oxobutanoic acid, PC(14:0/20:2(11Z,14Z)) was also among the top three metabolites capable of discriminating between groups with the highest accuracy. It was found that lipids and lipid-like molecules emerged as the predominant altered metabolites in individuals with new-onset sarcopenia compared to the control group. These findings are consistent with the study by Chi JL et al. (3) and Zhou J et al. (12). In our cohort study, we observed that the abundances of nine PC species [for example, PC(14:0/20:2(11Z,14Z)), PC (16:0/20:3(8Z,11Z,14Z)), and PC (18:1(11Z)/16:1(9Z))] were markedly reduced in subjects with sarcopenia compared to those in the control group. Nonetheless, a prior investigation exploring the association between resting phosphorus metabolites and skeletal muscle mass noted that older adults with sarcopenia exhibited heightened levels of PCs (34). The variability in PCs levels may be attributed to the presence of unsaturated double bonds. Wang et al. suggested that a decline in the levels of PCs with greater unsaturated double bonds could be closely associated with an elevated risk of sarcopenia in elderly populations (35). Diminished levels of PCs may potentially contribute to the buildup of enlarged mitochondria, which could become impaired and resistant to standard degradation processes via the autophagosomal/lysosomal pathway. This could result in heightened production of reactive oxygen species, thereby potentially exacerbating the aging process. However, additional evidence is necessary to substantiate this novel speculation.

### Relationship between the four enriched pathways and sarcopenia

In a previous study, it was suggested that metabolites showed remarkable enrichment of purine metabolism for KEGG pathway analysis (12), which supports our findings. Purine metabolism is integral to numerous physiological and pathological processes in mammals, encompassing the inflammatory response, oxidative stress reactions, and cancer (36, 37). The significance of inflammation and oxidative stress as pivotal factors in the pathogenesis of sarcopenia is widely acknowledged (38). KEGG pathway enrichment analysis indicated that hypoxanthine participated in this metabolic pathway. No study has investigated the role of parathyroid hormone synthesis in sarcopenia until now. Parathyroid hormone is a key regulator of calcium and phosphorus homeostasis. KEGG pathway enrichment indicated that 25(OH)D3 participates in this metabolic pathway. In our study, we observed higher plasma 25(OH)D3 levels in subjects with sarcopenia compared to healthy controls. This finding contradicts the findings of most studies, which have reported low 25(OH)D3 levels attributed to chronic inflammation or bacterial etiology (39). However, Zielińska et al. also found that 25(OH)D3 levels are elevated in inflammatory bowel diseases (40). Moreover, in an experimental model using C57BL/6 mice with induced muscle injury, the administration of excessive doses of 1α,25(OH)D3 or its intramuscular delivery did not show beneficial effects on muscle regeneration.it was observed to potentially have detrimental effects on satellite cell activity, ultimately compromising muscle fiber formation (41). In addition, the differentially expressed metabolite 25(OH)D3 also participates in the pathway of tuberculosis. The pathogenesis of sarcopenia in tuberculosis remains unknown. A thorough comprehension of the parathyroid hormone pathway’s involvement in tuberculosis can enhance our understanding of sarcopenia’s pathogenesis. In cancer, elevated levels of phosphocholine and total choline-containing compounds characterize the choline metabolite profile. Additionally, several studies have identified connections between choline metabolism in muscle tissue and both muscle protein synthesis and degradation. Therefore, further investigation into these metabolic pathways can advance our understanding of the pathological mechanisms underlying sarcopenia, thereby facilitating the development of more effective treatments.

### Strengths and limitations

This study represents one of the initial attempts to explore the connections between plasma metabolic signatures and the susceptibility to sarcopenia in Asian populations using an untargeted metabonomics platform. The untargeted metabonomics approach facilitates the identification of a wide array of metabolites, which will enhance our comprehension of the comprehensive landscape of crucial metabolic pathway alterations in sarcopenia. However, it is important to acknowledge several limitations of our study. Firstly, our findings were derived from a single cohort with a restricted number of cases and controls. Due to the small sample size, the analysis was not adjusted for multiple comparisons. Future research endeavors should incorporate more targeted omics analyses in other thoroughly characterized cohorts or larger validation cohorts to corroborate these findings, while implementing multiple comparison correction techniques. Second, further investigations are warranted to elucidate the precise molecular mechanisms underlying the observed results. Specifically, the role of metabolites such as PCs in sarcopenia remains to be evaluated, and mechanistic studies are imperative to delineate the exact contribution of these metabolites to the pathogenesis of sarcopenia. Additionally, we did not observe significant associations between amino acid metabolism pathways and sarcopenia, possibly due to the relatively small sample size. Sarcopenia is a complex condition influenced by various genetic, lifestyle, and environmental factors. A relatively small sample size may limit the ability to capture the full spectrum of metabolic changes associated with sarcopenia. Finally, the short follow-up time was also a major limitation of this study. Consequently, we plan to extend the follow-up duration in future research to enhance the power to evaluate the risk factors.

## Conclusion

In conclusion, we examined the connections between metabolic profiles and the susceptibility to sarcopenia utilizing LC-MS-based untargeted metabolomics methodologies. This investigation unveiled 62 early metabolic signatures and identified four metabolic pathways associated with sarcopenia, potentially enhancing the prognostication and prevention of sarcopenia in Chinese suburb-dwelling older adults. Notably, the top three metabolites, hypoxanthine, L-2-amino-3-oxobutanoic acid, and PC(14:0/20:2(11Z,14Z)), exhibit promise as novel plasma biomarkers for diagnosing sarcopenia. Nonetheless, these findings stem from a single, limited cohort, underscoring the necessity for future validation through robust, large-scale studies.

## Data availability statement

The original contributions presented in the study are included in the article/supplementary material. Further inquiries can be directed to the corresponding author.

## Ethics statement

The studies involving humans were approved by Ethics Committee of Shanghai University of Medicine and Health Sciences. The studies were conducted in accordance with the local legislation and institutional requirements. The participants provided their written informed consent to participate in this study.

## Author contributions

PH: Funding acquisition, Methodology, Formal Analysis, Writing – original draft. XC: Writing – original draft, Investigation, Validation. ZL: Validation, Writing – original draft, Methodology. YL: Data curation, Resources, Writing – review & editing. XY: Data curation, Writing – review & editing, Methodology, Software. PS: Data curation, Writing – review & editing, Formal Analysis. YZ: Data curation, Writing – review & editing, Methodology. HZ: Data curation, Methodology, Writing – review & editing, Investigation. SZ: Investigation, Writing – review & editing. XS: Writing – review & editing. QG: Writing – review & editing, Funding acquisition, Methodology, Supervision, Validation.
